# Shift-work and breastfeeding for women returning to work in a manufacturing workplace in Taiwan

**DOI:** 10.1186/s13006-022-00467-8

**Published:** 2022-04-07

**Authors:** Su-Ying Tsai

**Affiliations:** Department of Health Management, I-Shou University, Kaohsiung, Taiwan

**Keywords:** Work schedule, Shift work, Breastfeeding-friendly workplace, Breastfeeding rate, Female workers

## Abstract

**Background:**

Although breastfeeding-friendly workplaces are provided to promote an employed mother’s breastfeeding intention, few studies have explored breastfeeding intentions and behavior after a mother returns to work on a shift work or non-shift work schedule. To explore the impact of breastfeeding-friendly support on the intention of working mothers with different work schedules to continue breastfeeding, we conducted a survey at a female labor-intensive electronics manufacturer in Taiwan from August 2011 to April 2012.

**Methods:**

Female workers who met the inclusion criteria (maternity leave between January 2009 and January 2011) were invited to participate in the survey. A structured questionnaire survey was administered to 715 working mothers employed at an electronics manufacturing plant in Tainan Science Park in Southern Taiwan. The questionnaire content included female employee demographic characteristics, employment characteristics, continued breastfeeding behavior after returning to work, access to lactation rooms, and employee perception of the breastfeeding policy and support when raising their most recently born child.

**Results:**

A total of 715 employed mothers’ data were collected. Of the shift workers, 90.1% breastfed during maternity leave, but the breastfeeding rates after returning to work decreased to 21.5% for one to six months and 17.9% for more than six months. Of the non-shift workers, 87.6% breastfed during maternity leave and the breastfeeding rates after returning to work were 24.1% for one to six months and 34.6% for more than six months. Using a lactation room and taking advantage of breast-pumping breaks were significant factors for continuing to breastfeed one to six months after returning to work and more than six months after returning to work among shift workers and non-shift workers. In addition, among non-shift workers, a higher education level of the mother (odds ratio (OR) = 9.57) and partner support (OR = 4.89) had positive effects toward a mother continuing breastfeeding for more than six months after returning to work.

**Conclusions:**

Workplaces or employers should provide more support to encourage employed mothers to take advantage of the breastfeeding room and breast-pumping breaks, enhance the frequency of the usage of lactation rooms, and increase the rate of continued breastfeeding.

**Supplementary Information:**

The online version contains supplementary material available at 10.1186/s13006-022-00467-8.

## Background

Ever-increasing numbers of women in their childbearing years are employed outside the home [1, 2]. Although breastfeeding is known to be the best source of nutrition for infants [3, 4], many mothers experience barriers to maintaining a breastfeeding relationship with their infants upon returning to work and consequently terminate breastfeeding earlier than recommended or intended [5–7]. Many studies [8–11] have shown that returning to work early and working full-time are linked to lower odds of breastfeeding initiation and shorter breastfeeding durations, even for mothers with highly professional and autonomous work situations [8, 9]. The World Health Organization (WHO) recommends breastfeeding infants exclusively for six months, then continuing breastfeeding along with appropriate complementary foods until the child is at least two years of age [12]. Even though Taiwan’s national policy promotes exclusive breastfeeding, more recent surveillance showed that breastfeeding indicators stagnated or even decreased. A recent survey of breastfeeding among community mothers in southern Taiwan revealed a decline in the prevalence of breastfeeding, and overall, only 40.1 and 29.3% who started breastfeeding still breastfed their infants at one and two months after childbirth, respectively [13]. Another survey examining the breastfeeding trends at six months postpartum during 2011–2016 in a national survey in Taiwan among postpartum women revealed that the rates of exclusive breastfeeding at six months postpartum declined from 24.5 to 14.8% [14]. Exclusively breastfeeding while working is more difficult for working women if workplace support is minimal, and inadequate breastfeeding facilities in the workplace are a risk factor for breastfeeding discontinuation [15].

To meet the WHO recommendation and increase the breastfeeding rate after mothers return to work, a breastfeeding-friendly workplace policy, including the provision of lactation rooms, breast-pumping breaks for employees to express breast milk for children, and a mothers' support system that includes healthcare professionals during the postpartum period are critical elements for improving the rates of breastfeeding duration and exclusivity [16–19]. In Taiwan, the law stipulates that employers must provide eight weeks of maternity leave for female employees. The government encourages companies or industries to provide breastfeeding support services, such as breast-pumping breaks and lactation rooms. Employees must bring their own breast pumps. Although such breastfeeding-friendly policies have been established to provide and promote employed mother’s breastfeeding intention, very little research has explored infant-feeding intentions and behaviors of mothers with different work schedules, such as shift and non-shift (9-to-5 work schedule) workers, as well as their breastfeeding enablers and obstacles, given a breastfeeding-friendly workplace.

Sustaining breastfeeding requires time and commitment on behalf of the mother. Mothers often need to combine breastfeeding with returning to work. Studies have demonstrated that the type of a mother’s employment affects her experience of combining work and breastfeeding. Women in management and professional occupations may have much more access to support when returning to work compared with women in temporary employment or in service industries who report very low levels of support [20]. With regard to non-standard work schedules, mothers who work evening or night shifts generally have lower educational attainment, work fewer hours per week, and earn less than those who work standard hours, and the work schedule itself (hours) is a key factor influencing maternal and child wellbeing, family function, and breastfeeding behavior [21]. A previous study found that a rotating night shift does not affect breast milk volume [22]. Some studies report that women are hesitant to ask for adjustments to their schedule to enable breastfeeding for fear that it will be interpreted as a lack of commitment to the workplace and a privileging of their personal life [23], and that women report feeling self-conscious at work and viewed as less professional or uncommitted if they combine breast milk expression with the return to work [24]. Feeling self-conscious when breastfeeding negatively affects exclusive breastfeeding for the recommended duration [25]. These feelings of misunderstanding among employed mothers influence breastfeeding behavior and intention after returning to work and are worthy of deeper investigation.

In this present study, we conducted a survey among mothers employed in a female-dominated workforce electronics manufacturing company in Taiwan to explore the intention to continue breastfeeding after returning to work among employed mothers who are and are not shift workers, and the associated factors of breastfeeding behavior in a breastfeeding-friendly workplace. The findings of the present study will provide important information to occupational and environmental health nurses regarding the barriers to continuing breastfeeding and the implementation of breastfeeding-supportive services for mothers with different types of work schedules.

## Methods

### Sample and setting

This was a retrospective survey of a breastfeeding-friendly workplace and intention to continue breastfeeding after returning to work among employed mothers in Taiwan and was conducted from 1 August 2011 to 30 April 2012. The research setting was Company C, a large highly labor-intensive electronics manufacturer in the Tainan Science Park in Southern Taiwan, which is one of Taiwan’s largest areas for electronics manufacturers. This company has more than 20,000 employees, 45% of whom are female, and there are ten manufacturing plants. Each plant provides at least four lactation rooms for working mothers, with the largest plant providing 11 lactation rooms**.** The research methodology of this study is reported elsewhere [18, 19].

Company C was selected for the following reasons. First, it is one of a number of companies that have received funding from the Department of Health to establish lactation rooms in its factories, and therefore provides lactation rooms and breast-pumping breaks for working mothers. Second, this company has many female employees. Moreover, the female employees are office workers or clean-room workers (a room that is maintained virtually free of contaminants used for the production of precision parts for electronic equipment). Office workers have higher education and fewer shift work positions than clean-room workers, and generally work about eight hours a day, but their positions encompass specific job responsibilities. By comparison, clean-room workers generally work 12-h shifts. Their jobs are inconvenient and inflexible because they must remove and don clean-room suits when leaving and returning to their workstation. Therefore, we were able to observe the association between different work schedule conditions and intention to continue breastfeeding. The researcher inquired about the willingness of this company to participate in the study by first sending an explanatory letter about the research project, and then visiting the employee health management department director of the company to explain the purpose of the research. After receiving consent from the employee health management department, occupational and environmental health nurses helped distribute and collect the employed mothers’ paper-based self-reported questionnaires. The questionnaire was distributed to 981 female employees who had recently taken maternity leave between January 2009 and January 2011, as recorded by the human resources department. Female workers who met the inclusion criteria were invited to participate in the survey. A total of 715 valid questionnaires were collected, providing a response rate of 72.9%. The study was approved by the Institutional Review Board of I-Shou University (date of IRB approval: 2 September 2011).

### Data collection

Hard copies of the self-reported questionnaires were used to collect data on female employees’ demographic and employment characteristics, continuing breastfeeding behavior after returning to work, access to lactation room types, and employees’ perception of the breastfeeding-friendly policy and support when raising their most recently born child. Mean time required to complete the survey was 15 min.

### Demographic and employment characteristics

Participants’ self-reported demographic and employment characteristics were assessed. A demographic inventory was used to gather data on age, education, husband’s education, and child information. Level of education was used as a proxy measure for social class and categorized as follows: (1) high school or below or (2) college or above.

Employment characteristics were collected, including worksite (office vs clean room), shift work (“Did you do shift work after you returned to work?” yes / no), and work hours per day (eight hours a day or 9 − 14 h a day).

### Breastfeeding after return to work

Working mothers were defined as ‘continuing breastfeeding’ if they continued to breastfeed for at least one month after returning to work. ‘Any breastfeeding’ was defined as the child receiving any type of breast milk feeding, no matter if combined or not with other diets. In this study, we adopted this definition to collect continuing breastfeeding behavior among employed mothers. Participants were asked, “Did you continue to breastfeed after returning to work (yes / no) and how long did you continue to breastfeed? (survey employed mothers’ breastfeeding months)” In Taiwan, all companies must provide eight weeks of maternity leave for female employees. Hence, we also assessed the breastfeeding behavior during maternity leave and participants were asked, “Did you breastfeed your baby during maternity leave (yes / no)?”.

### Access to lactation room type and employees’ perception of breastfeeding-friendly policy and support

In our study, all lactation rooms in this company contain a table, chair, sink, electrical outlets, and refrigerator, and employers allow working mothers to have two breast-pumping breaks each day with each break lasting no more than 30 min. The researcher visited the plants and recorded observations about the facilities and lactation rooms, which were classified into two types: breastfeeding rooms with dedicated space vs shared space: those without dedicated space; only curtain separators. Working mothers pumping in shared space meant that working mothers pumped in a public health center room without dedicated space. In these rooms, only curtain separators were available in a space used mainly for other purposes, such as a public health center for acute trauma or injuries.

To understand an employee’s perception of the breastfeeding-friendly policies in the workplace, participants responded to the following questions: “What kind of lactation room was available in your workplace (dedicated space / shared space)”, “Were you aware of the pumping break policy?” (yes / no), “Did you ever use the pumping break policy after returning to work?” (yes / no), and “Did you feel embarrassed if you used breast-pumping breaks?” (yes / no). Moreover, to assess employees’ perceptions of workplace breastfeeding support, participants were asked, “After returning to work, did your colleagues, supervisor, environmental health nurses, and partner / husband encourage you to use breast-pumping breaks?” (yes / no), “Do you agree that taking breast-pumping breaks will reduce a working mother’s work efficiency?” (yes / no), and “Do you feel that taking breast-pumping breaks will affect your supervisor’s assessment of your performance?” (yes / no).

### Statistical analysis

This study separately explored the associated factors of continuing to breastfeed one to six months after returning to work and more than 6 months after returning to work among shift workers and non-shift workers. The primary independent variables of interest were demographics (age and working mother’s education level), employment characteristics (worksite, work hours per day), access to type of lactation room, breastfeeding-friendly policy (awareness of breast-pumping breaks, using lactation room and using breast-pumping breaks) and support (encouragement from colleagues, supervisors, environment health nurses, and partner / husband). In this study, we focused on breastfeeding behavior after returning to work among working mothers. Because the previous study [4–6] revealed that many mothers experienced barriers to maintaining a breastfeeding relationship with their infants upon returning to work and consequently terminated breastfeeding earlier than recommended or intended. The dependent variables in this study were continuing to breastfeed after returning to work. Working mothers were defined as continuing breastfeeding if they continued for at least one month after returning to work from maternity leave. Hence, working mothers who did not breastfeed at the beginning of maternity leave and breastfed for less than one month after returning to work were categorized as not continuing to breastfeed after returning to work and were treated as a reference group in the logistic regression analyses. Moreover, the WHO recommends breastfeeding infants exclusively for six months, then continuing breastfeeding along with appropriate complementary foods until the child is at least two years of age. This study focused on breastfeeding behavior after returning to work, so we collected data on whether mothers continued breastfeeding six months after returning to work (i.e., until eight months of infant age). All analyses were performed using Statistical Analysis System (SAS 6.12; SAS Institute, Cary, NC) software.

Participants’ profiles, breastfeeding encouragement from peers (colleagues, supervisors, environment health nurses, and partner / husband), and employed mother’s perceived feelings of using breast-pumping breaks among mothers with and without shift work were reported. The association of demographic and employment characteristics and breastfeeding-friendly policy on continuing to breastfeed after returning to work were estimated using chi-square tests. A *P* value of less than 0.05 was considered statistically significant. To determine whether the independent variables were associated with continuing to breastfeed after returning to work, multiple logistic regression analyses were used to identify independent variables that were independently associated with continuing to breastfeed for at least six months after returning to work and continuing to breastfeed for more than six months after returning to work, respectively. The odds ratio was calculated for each independent variable in the logistic models, and 95% confidence intervals (CIs) were calculated using maximum likelihood methods.

## Results

In the present study, we analyzed the data of 715 employed mothers. Characteristics of the study sample are shown in Table 1**.** A comparison of characteristics between mothers with and without shift work revealed that all variables differed significantly between the two groups (*p* < 0.05), except for the awareness of the breast-pumping breaks policy (*p* = 0.0689) and breastfeeding during maternity leave (*p* = 0.29).

**Table 1:** Characteristics of study sample (*n* = 715)

Variables		Shift work (*n* = 334)		Non-shift work (*n* = 381)		*P*-value
		**n**	**%**	**n**	**%**	
Age (years)	20–29	99	29.64	72	18.89	< 0.01
	≥ 30	235	70.36	309	81.11	
Education	College and above	199	59.58	313	82.15	< 0.01
	Below college level	135	40.42	68	17.85	
Worksite	Clean room	253	75.74	67	17.58	< 0.01
	Office	81	24.26	314	82.42	
Work hours per day	8	7	2.09	112	29.39	< 0.01
	9 − 14	327	97.91	269	70.61	
Access to lactation room	Share space	328	98.20	280	73.49	< 0.01
	Dedicated space	6	1.80	101	26.51	
Using lactation room	Yes	145	43.41	274	71.91	< 0.01
	No	189	56.59	107	28.09	
Awareness of breast-pumping breaks policy	Yes	260	77.84	274	71.91	0.06
	No	74	22.16	107	28.09	
Using 2 breast-pumping breaks per day	Yes	91	27.24	168	44.09	< 0.01
	No	243	72.76	213	55.91	
Breastfeeding during maternity leave	Ever breastfed	301	90.11	334	87.66	0.29
	Never breastfed	33	9.89	47	12.34	
Continue to breastfeed after returning to work (breastfed for at least 1 month)	Yes	132	39.52	224	58.79	< 0.01
	(1 − 6 months)	(72)	(21.55)	(92)	(24.14)	0.01
	(> 6 months)	(60)	(17.96)	(132)	(34.64)	
	No	202	60.47	157	41.20	

Comparison of breastfeeding encouragement from peers and the employed mother’s feelings about taking advantage of breast-pumping breaks between mothers employed to work shifts and non-shifts is shown in Table 2. Encouragement by peers, including colleagues, supervisor, environmental health nurses, and partner / husband, to continue lactation after returning to work was not significantly different between the two groups. Shift workers were more likely than non-shift workers to feel that taking two breast-pumping breaks can reduce a mother’s work efficiency (56.28 vs. 49.60%; *p* < 0.01).

**Table 2:** Comparison of peer encouragement and employed mother’s perceived feeling using breast-pumping breaks between working mothers with and without shift work

Variables	Total (%)	Shift work (%)	Non-shift work (%)	*P*-value
Using breast-pumping breaks				
Colleagues encourage me to use breast-pumping breaks	76.64	75.15	79.95	0.37
Supervisor encourages me to use breast-pumping breaks	59.44	59.28	59.84	0.93
Environmental health nurses encourage me to use breast-pumping breaks	66.60	64.37	68.50	0.24
Partner/husband encourages me to use breast-pumping breaks	80.55	79.94	81.10	0.69
A feeling of embarrassment using breast-pumping breaks	32.45	32.03	32.80	0.82
A feeling of two breast-pumping breaks can reduce a working mother’s work efficiency	51.05	56.28	49.60	0.01
A feeling that taking 2 breast-pumping breaks will influence a supervisor’s evaluation of performance	45.17	48.50	43.30	0.09

Association between continued breastfeeding behavior and associated factors among employed mothers with and without shift work are shown in more detail [see Additional file 1].

The results of the logistic regression analysis evaluating independent factors of continuing breastfeeding after returning to work during the first six months and continuing to breastfeed for more than six months among the two groups are shown in Table 3. Using a lactation room and taking advantage of breast-pumping breaks were significant factors for continuing to breastfeed for mothers returning to work between one to six months and after more than six months among shift workers. Among non-shift workers, significant factors for a mother’s intention to continue breastfeeding during the first six months after returning to work were using a lactation room (OR = 8.37; 95% CI 3.28, 21.33) and taking advantage of breast-pumping breaks (OR = 18.34; 95% CI 6.31, 53.28); significant factors that predict a mother’s intention to continue breastfeeding for more than 6 months after returning to work were higher education (OR = 9.57; 95% CI 2.32, 39.47), using a lactation room (OR = 5.33; 95% CI 1.98, 14.33), taking advantage of breast-pumping breaks (OR = 55.48; 95% CI 16.88, 182.33), and encouragement by the partner / husband (OR = 4.89; 95% CI 1.07, 22.38).

**Table 3:** Association between intention to continue breastfeeding and associated factors among employed mothers by multiple logistic regression

Variables	Continue to breastfeed after returning to work with shift work		Continue to breastfeed after returning to work without shift work	
	**between 1–6 months**	** > = 6 months**	**between 1–6 months**	** > = 6 months**
	**AOR**^**a**^** (95%CI)**	**AOR**^**a**^** (95%CI)**	**AOR**^**a**^** (95%CI)**	**AOR**^**a**^** (95%CI)**
Age (< 30 vs ≧30)	1.63 (0.62 − 4.27)	0.94 (0.27 − 3.31)	1.56 (0.62 − 3.90)	0.59 (0.15 − 2.26)
Education (≧ College vs ≦ High school)	0.98 (0.39 − 2.49)	1.13 (0.38 − 3.40)	1.74 (0.67 − 4.48)	9.57 (2.32 − 39.47)
Worksite (clean room vs office)	1.45 (0.51 − 4.09)	2.19 (0.63 − 7.61)	0.76 (0.29 − 1.98)	0.57 (0.18 − 1.80)
Work hours per day (8 vs 9 − 14)	0.36 (0.06 − 2.12)	1.36 (0.22 − 8.31)	1.43 (0.64 − 3.19)	2.43 (0.98 − 6.01)
Using lactation room	50.33 (18.55 − 136.52)	76.87 (18.85 − 313.49)	8.37 (3.28 − 21.33)	5.33 (1.98 − 14.33)
Awareness of breast-pumping breaks policy	0.62 (0.22 − 1.73)	1.39 (0.38 − 5.07)	2.07 (0.90 − 4.73)	0.86 (0.34 − 2.17)
Using breast-pumping breaks	5.20 (1.78 − 15.21)	4.10 (1.16 − 14.43)	18.34 (6.31 − 53.28)	55.48 (16.88 − 182.33)
Colleagues encourage me to use breast-pumping breaks	1.80 (0.41 − 7.99)	4.05 (0.67 − 24.21)	3.85 (0.98 − 14.23)	1.97 (0.42 − 9.30)
Supervisor encourages me to use breast-pumping breaks	0.77 (0.16 − 3.60)	0.34 (0.07 − 1.68)	0.29 (0.09 − 1.46)	0.42 (0.11 − 1.58)
Environmental health nurses encourage me to use breast-pumping breaks	1.01 (0.22 − 4.53)	1.77 (0.37 − 8.40)	1.99 (0.66 − 5.95)	1.19 (0.28 − 4.92)
Partner encourages me to use breast-pumping breaks	0.77 (0.17 − 3.51)	0.83 (0.09 − 7.06)	1.28 (0.38 − 4.34)	4.89 (1.07 − 22.38)

^a^*AOR* adjusted odds ratio and adjusted for all variables in the model

## Discussion

Returning to work while still breastfeeding is challenging. In the present study, 90.1% of shift workers and 87.7% of non-shift workers initiated breastfeeding at the beginning of maternity leave, but the rate of continuing to breastfeed rapidly decreased after returning to work. The rate of continuing to breastfeed for at least one month after returning to work was only 39.5 and 58.8% for shift workers and non-shift workers, respectively. In addition, continuing breastfeeding decreased at a significantly more rapid rate among shift workers compared with non-shift working mothers (*p* = 0.01). The withdrawal rates of breastfeeding after returning to work until six months later was 72.2% (90.1–18.0%) among shift-working mothers, and there are 52.0% (87.7 − 34.6%) withdrawal rates of breastfeeding after returning to work until six months later among non-shift workers. These withdrawal rates among the two groups of employed mothers were still huge, even if lactation rooms and breastfeeding-friendly policies (e.g., daily breast-pumping breaks) were available in this study workplace. Previously, breastfeeding was generally thought to be an individual's decision and the sole responsibility of a woman to succeed, demonstrating maternal devotion. One study [26] mentioned multifactorial determinants of the decision to breastfeed — the need for supportive measures at many levels, from legal and policy directives to social attitudes and values, women's work and employment conditions, and health-care services to enable women to breastfeed. In addition to work-time breaks and on-site rooms for breastfeeding in the workplace, adequate delivery of relevant interventions and multi-dimensional problem-solving, such as by addressing fatigue, practicality, and intensity, may rapidly enhance breastfeeding practices.

One study [27] suggested that dedicated lactation specialists may play a role in providing education and support to pregnant women and new mothers wishing to breastfeed to improve breastfeeding outcomes. The employer can positively develop and improve postpartum support programs incorporating lactation consultants and lactation counselors. A recent study [28] demonstrated that self-efficacy is an important predictor for breastfeeding duration; hence, workplaces may help bolster women's self-efficacy by providing an environment or atmosphere that is supportive for breastfeeding working mothers. In a quasi-experimental study in Japan [29], a program intervention involving a 90-min breastfeeding class, a pamphlet, a newsletter, and email consultation was associated with a significant increase in breastfeeding continuation rates three months after returning to work. Similarly, due to the reinforcing effect of intention and self-efficacy, breastfeeding support should focus on helping first-time mothers to succeed as well as identifying second-time mothers with low self-efficacy and additional need for support [30]. A study in Greece evaluated the efficacy of a 12-month intervention for mothers before and after childbirth and revealed that continuous long-term midwife-led education and support, and maternal mental well-being are associated with increased exclusive and any breastfeeding duration [31]. Creating an enabling breastfeeding environment and atmosphere is not just about providing a breastfeeding room or introducing supportive policies (e.g., two breast-pumping breaks); it is also about actively seeking to provide support or assisting in providing successful breastfeeding experiences and offering education programs such as a long term midwife-led breastfeeding support and psychosocial support for employed women.

The type of employment a woman is engaged in can affect her experience of combining work and breastfeeding and her feelings of self-consciousness and embarrassment when expressing breast milk [32]. Women in management and professional occupations have much more access to support when they return to work compared with women in temporary employment who report very low levels of support [33]. Women are hesitant to ask for adjustments to their schedule to enable breastfeeding for fear that it will be interpreted as a lack of commitment to the workplace and a privileging of their personal life [23]. Our data revealed that mothers employed as shift workers had higher rates than non-shift workers of feeling that taking two breast-pumping breaks reduces a mother’s work efficiency. Although awareness of the company policy to provide two breast-pumping breaks did not differ between clean-room and office-worksite working mothers, working mothers with shift work used the lactation room and breast-pumping breaks less often than non-shift workers. Besides, more shift-working mothers worked in the clean room than non-shift–working mothers (75.7 vs. 17.6%). Mothers working in the clean room had more difficulties using the breast-pumping breaks than those at office worksites because workers in the clean room at the electronics manufacturing plant needed time to take off and don their clean-room suits, implying that an inconvenient work environment is an important barrier to breastfeeding among working mothers. Moreover, the fast-speed schedules on the production line of the electronics industry and the relatively inflexible assembly work make it difficult for female employees to rest to use the breast-pumping breaks. Only 2.1% of shift workers averaged work eight hours per day; 97.9% of shift workers had a high workload of average work 9 − 14 h per day, which is a high-burden employment for shift-working mothers.

In our study, non-shift workers had a higher rate of high-level education than shift workers. It is possible that working mothers with high-level education have more control over their environment and schedules, and are able to combine breastfeeding and working more successfully than clean-room working mothers. One study [34] examined staff employed in a diverse range of clinical and non-clinical occupations, including doctors, nurses, allied health, population health, administration, management, information technology, and domestic services, and found that clinical staff may have less flexibility in their daily workload due to patient demands, which could impact their ability to take regular lactation breaks; hence, flexible work options and lactation breaks were identified as the main factors that facilitate breastfeeding at work [34]. Many similar studies highlighted the need for a support system that encourages flexible working hours as a key determinant of breastfeeding initiation and continuation [34–36]. In our study, the use of a lactation room and breast-pumping breaks were significant predictors of continued breastfeeding for between one to six months and more than six months after returning to work among the two groups after adjusting for other variables. Hence, employers, supervisors, or environmental health nurses may assist employed mothers by, for example, providing a lower workload for shift workers who want to continue breastfeeding or adjusting the workload in the office, to promote a balance between work and a continuous breastfeeding experience. These are all important support actions that can help mothers use the breast-pumping break time at work and assist shift-working mothers to continue breastfeeding after returning to work.

In our study, higher education and encouragement from partner / husband to use breast-pumping breaks were significant predictors of continued breastfeeding for more than six months after returning to work among non-shift working mothers. Previous studies [36–39] reported that the decision to breastfeed and the likelihood of early initiation of breastfeeding was associated with a higher-level educational status of the mother. Women employed as professionals breastfeed longer than other working mothers [33]. In our study, 82.2% of non-shift workers had a higher education level (college and above), and were thus regarded as white-collar workers with potentially more control over their environment and schedules, allowing them to combine breastfeeding and working more successfully than shift-working mothers. In addition, partner support was a significant predictor of continued breastfeeding for more than six months after returning to work among non-shift working mothers, revealing that partner support may enhance maternal self-efficacy for breastfeeding. In a Japanese study, family support and a high paternal education level were crucial for enabling working mothers to practice exclusive breastfeeding [40]. Another study also showed that father’s knowledge in exclusive breastfeeding can, in one way, significantly enhance mother’s knowledge by sharing and, in another way, can enhance his own attitude to offer different support to his partner / wife that induces the chances of exclusive breastfeeding practices by mothers [41]. Future research should assess the current state of mutual support necessary for breastfeeding from the perspective of the wife as the support recipient and the partner / husband as a support provider.

There are some limitations to our study. First, this study was cross-sectional in design; therefore, only associations could be evaluated, not causation. Second, assessment of predictors adopted a dichotomized classification, which was simplistic, and predictor measurements mainly relied on self-report, which might have biased the results. Third, a selection bias due to non-response was inevitable. Fourth, the participants were recruited over ten years ago and as such there could be recall bias with the self-report questions. This could easily affect the internal validity of the study. In addition, we suggested future studies on this topic should use valid and reliable tools to measure breastfeeding intention rather than self-created questions to enhance the scientific integrity. Nevertheless, using a lactation room and taking advantage of breast-pumping breaks were significant predictors of continuing to breastfeed for between one to six months and for more than six months after returning to work among shift workers and non-shift workers. In addition, a higher education level of the mother and partner support had a positive effect on continuing to breastfeed for more than six months after returning to work among non-shift workers.

## Conclusion

The findings of this study suggest that to truly be a breastfeeding-friendly workplace environment, workplaces and employers should provide more support for employed mothers to take advantage of the breastfeeding room and breast-pumping breaks through flexible work adjustments or reduced workload, which will increase the rate of continued breastfeeding.

## Supplementary Information

**Additional file 1.** Associationbetween continued breastfeeding behavior and associated factors among employed motherswith and without shift work.

## Data Availability

All data generated or analyzed during this study are included in this published article.

## Abbreviations

WHO: World Health Organization
