# Supplementary material for: Shift-work and breastfeeding for women returning to work in a manufacturing workplace in Taiwan
Source: Int Breastfeed J. 2022 Apr 7;17:27. doi: 10.1186/s13006-022-00467-8 (PMC8991565; doi:10.1186/s13006-022-00467-8)
Supplement: Supplementary file 1 — Additional file 1. Associationbetween continued breastfeeding behavior and associated factors among employed motherswith and without shift work. [file 13006_2022_467_MOESM1_ESM.docx]

Association between continued breastfeeding behavior and associated factors among employed mothers with and without shift work.

| Variables |  | | | Continue to breastfeed after returning to work | | | | | | | | | | | | | | | |
| --- | --- | --- | --- | --- | --- | --- | --- | --- | --- | --- | --- | --- | --- | --- | --- | --- | --- | --- | --- |
|  |  |  |  | with shift work (n=334) | | | | | | | | without shift work (n=381) | | | | | | | |
|  |  |  |  | No (n=202) | | ≦6 months (n=77) | | > 6 months (n=55) | | P-value |  | No (n=157) | | ≦6 months (n=107) | | > 6 months (n=117) | | P-value | |
| Worksite | Clean room  Office | | | 158 (78.21)  44 (21.79) | | 53 (68.8)  24 (31.2) | | 42 (76.36)  13 (23.64) | | 0.26 | | 40 (25.47)  117 (74.53) | | 14 (13.08)  93 (86.92) | | 13 (11.11)  104 (88.89) | | <0.01 | |
| Work hours per day | 8  9+ | | | 6 (2.94)  196 (97.03) | | 0 (0)  77 (100) | | 1 (1.81)  54 (98.19) | | 0.29* | | 48 (30.57)  109 (69.43) | | 32 (29.90)  75 (70.10) | | 32 (27.35)  85 (72.65) | | 0.83 | |
| Access to lactation room | Share space  Dedicated space | | | 196 (97.0.)  6 (2.94) | | 77 (100)  0 (0) | | 55 (100)  0 (0) | | 0.18* | | 103 (65.60)  54 (34.39) | | 80 (74.76)  27 (25.24) | | 97 (82.90)  20 (17.10) | | <0.01 | |
| Using lactation room | Yes  No | | | 23 (11.38)  179 (88.61) | | 70 (90.90)  7 (9.10) | | 52 (94.54)  3 (5.46) | | <0.01 | | 66 (42.04)  91 (57.96) | | 99 (92.52)  8 (7.48) | | 109 (93.16)  8 (6.84) | | <0.01 | |
| Awareness of breast pumping breaks policy | Yes  No | | | 149 (73.76)  53 (26.42) | | 63 (81.81)  14 (18.19) | | 48 (87.27)  7 (12.73) | | 0.06 | | 91 (57.96)  66 (42.04) | | 87 (81.30)  20 (18.70) | | 96 (82.05)  21 (17.95) | | <0.01 | |
| Using breast pumping breaks | Yes  No | | | 11 (5.44)  191 (94.56) | | 44 (57.14)  33 (42.86) | | 36 (65.45)  19 (34.55) | | <0.01 | | 6 (3.82)  151 (96.18) | | 70 (65.42)  37 (34.58) | | 92 (78.63)  25 (21.37) | | <0.01 | |
| Colleagues encourage me to use breast-pumping breaks | | Yes  No | 138 (68.31)  64 (31.69) | | 65 (84.41)  12 (15.59) | | 48 (87.27)  7 (12.73) | | <0.01 | | 107 (68.15)  50 (31.85) | | 93 (86.91)  14 (13.09) | | 97 (82.90)  20 (17.10) | | <0.01 | |  |
| Supervisor encourages me to use breast-pumping breaks | | Yes  No | 111 (54.95)  91 (45.05) | | 52 (67.53)  25 (32.47) | | 35 (63.63)  20 (36.37) | | 0.12 | | 85 (54.14)  72 (45.86) | | 71 (66.35)  36 (33.65) | | 71 (60.68)  46 (39.32) | | 0.13 | |  |
| Environmental health nurses encourage me to use breast-pumping breaks | | Yes  No | 120 (59.40)  82 (40.60) | | 55 (71.42)  22 (28.58) | | 40 (72.72)  15 (27.28) | | 0.06 | | 94 (59.87)  63 (40.13) | | 81 (75.70)  26 (24.30) | | 86 (73.50)  31 (26.50) | | <0.01 | |  |
| Partner/husband encourages me to use breast-pumping breaks | | Yes  No | 147 (72.77)  55 (27.23) | | 69 (89.61)  8 (10.39) | | 51 (92.72)  4 (7.28) | | <0.01 | | 113 (71.97)  44 (28.03) | | 95 (88.78)  12 (11.22) | | 101 (86.32)  16 (13.68) | | <0.01 | |  |

* Fisher's Exact Test
